# Correlation of Physical and Vital Force; Clinically Illustrated

**Published:** 1871-05

**Authors:** Z. C. McElroy

**Affiliations:** Zanesville, Ohio


ARTICLE III.—*Correlation of Physical and Vital Force; Clinically Illustrated.* By Z. C. McELROY, M.D., Zanesville, Ohio.

(A Paper read to the Muskingum County Medical Society, April 7, 1871.)

Questions of science and fact are not often settled by after-dinner addresses. And for their utterances on such occasions, gentlemen are very properly not held to very strict account.

But it is, nevertheless, to be regretted that those whose positions in life give to their utterances on all public occasions an *ex cathedra* importance, should ever discuss questions of science and fact as partisans of particular conclusions, in the absence of positive demonstration.

Prof. Gunn,* at the recent commencement of Rush Medical College, discussed at some length, and in no catholic and scientific spirit, it seems to me, the correlation of the physical and vital forces; but rather as a partisan that no such correlation exists in fact; and felicitously introduces the Indian story of turkey and crow, to make more dramatic the position the parties occupy, for and against it, in the controversy now in progress.

"Brudders, 'pears to me not one of ye hab on any part of de Christin harness but de brechin, for ye don't do nothing but hold back," said the colored preacher to the congregation with whom he had been laboring ineffectually for a revival. That, it seems to me, is Prof. Gunn's position in science. Or, it may be, in regard to the correlation of the physical and vital forces, he occupies that of the Irishman towards oysters as food, "he did not like them, and by his modesty never would." So, on that occasion, Prof. Gunn would not allow physical and vital force to be correlated, and seemed determined they never should be. But his fixing it up was, perhaps, like the fixing of Mr. Smith's old clock, it would not stay fixed.

Whether the so-called vital and physical forces are correlated in the human body, is a question of fact, not of fancy, and must be decided, as all other questions, by the facts for or against it, that is, demonstration.

* Chicago Medical Journal, March, 1871.

Stripped of its technical clothing and surroundings, the naked question may be stated as follows:

"Are the phenomena presented by the human body, mechanical, chemical, thermal, sensory, emotional, intellectual, and psychological, ultimately due to the operation of the ordinary physical forces, or are they due to a peculiar vital force manifested nowhere else in the world than in living beings?"

Some years since I took up this question for study, altogether indifferent as to the conclusions to be reached, so that they should be ultimate truth. I did not study it to satisfy my curiosity, but to define more clearly my duties and power in prescribing for the sick. And the purpose of this paper is to map, in outline, the steps by which a satisfactory conclusion to my mind was reached. As I was better acquainted with my own body than that of any other person, it was constantly referred to in the study. And I brought to bear in the investigation what I think was a tolerably full acquaintance with the accumulated facts of life, physiological, pathological, dynamic, and therapeutic.

As a starting point, it was noticed, particularly, that I was located on the surface of the earth, which was whirling its way through space in its annual circuit round the sun, at the rate of many thousand miles per hour; and therefore not occupying any one point in space. any time appreciable to my senses. The nearest planetary neighbor was some two hundred and thirty seven thousand miles distant; while the sun, the center of our planetary system, was distant some ninety odd millions of miles. All that is certainly known to be received on the earth from either the central luminary, sister planets, or remoter stars, is the light and heat of the sun, direct and indirect.

Looking over the earth's surface it was observed that animal life existed in the precise ratio of the amount of light and heat of the sun received at different points of the earth's surface, with a soil capable of sustaining vegetable growth and life. In other words, life only existed under certain physical conditions. It was therefore evident to my mind that the sun's rays and life had some intimate relationship to each other—were, in fact, cause and effect, so to speak, palpably linked together. But how? The rays of the sun might fall on the most delicate substance for days, weeks, months, or even years, with no direct traces of any effects, except such as were attributed to "the weather." The "imponderable elements" of natural philosophy and chemistry—light, heat, electricity, magnetism, galvanism, gravity, chemical affinity, or ozone, it seemed a hopeless task to connect with life. Soil and sunlight, and the human body! how unlike, and how seemingly impossible that they are in matter and force the same!

Yet that is precisely the Creator's account. "And the Lord God FORMED man of the dust of the ground, and breathed into his nostrils the breath of life, and man became a living soul." (Gen. ii. 7.) The first, and principal, act of creation, was making the forms of the body; the second, through the nostrils communicating life, or motion to the form; and the result, man became a living soul. And this account of the genesis of the human body is not only literally, but scientifically, correct to-day. The forms of the body are built up during intra-uterine life, and motion communicated through the nostrils immediately after birth to the form. And the same order in the succession of events are still necessary, and observed in its perpetuation; the female furnishing the material, the male impressing the materials with the forms, and the result, a living soul! And the whole of the proceedings still take place on earth, with materials common to earth, and by the earth's forces. For the materials of the ovum supplied by the female, as well as that supplied by the male, in the act of reproduction, must necessarily be from the food eaten by them respectively, which, in families, is from one common table; and all directly or indirectly from the vegetable world; and ultimately from inorganic materials. And at last, sunlight and soil.

Early education, however, had impressed the idea on my mind that, in some way, not definitely understood, human bodies had, in part, an extra-terrestrial origin; that man was the "lord of creation," a vastly superior being to any other forms of organic life around him; in fact, that all other animal and vegetable life existed merely to minister to his wants, necessities and pleasures. True, there were some ugly facts in the way of such conclusions; as, for instance, that the flesh of most of the inferior animals could be, and were, used as part of his food. And it was tolerably certain that human bodies were constructed out of the food eaten. But that which staggered me most, was that the continuance of this most elevated, or celestial force, *i. e.*, life, or vital, was at the mercy of so many physical contingencies. Thus, life could be terminated by water, drowning, and yet a certain proportion was necessary for its continuance; combustion, heat, and yet a certain proportion, like water, was a necessity; certain mechanical processes, as falls, blows, bullets and bombs; starvation; chemical combinations of matter; animal, as the virus of serpents, insects, etc.; vegetable, as opium, nux vomica, etc.; and inorganic, as salts, or oxides of arsenic, copper, lead, etc. The conclusion, then, that the importance of any so-called vital force, peculiar to the human body, and controlling its physical organization, has been somewhat exaggerated, seems well nigh irresistible in the presence of such an array of facts, to which few or none of us can be wholly strangers. It will be seen, in the sequel, the question "has it any existence?" is still more pertinent.

Then I was confronted in the literature and teachings of the profession with a list of several hundred specifically distinct entities of disease; and from thirty to forty distinct classes of medicines! sufficient to appal the bravest and best cultivated intellect, and to smother at once any hope that order could be established in the midst of such a chaos.

The first, because the most obvious conclusion reached was, that in all the varied and apparently incomprehensible phenomena of organized life, nothing happened by chance or accident, but in obedience to fixed and unchangeable law. Then I wrote down as the first general principle, that "the reign of law was supreme in organic and inorganic natures." The next safe ground reached was, that neither matter nor force could be annihilated; that as one mode of force disappeared, it was merged into some other mode of force; and that matter was equally persistent. That was written down as "the correlation and conservation of force, and continuity of matter."

The next points for decision were "By what means do I, as an organized existence, or being, know that there are such things as matter and force? How do I know that there is light, or sound? How and by what means are the faculties of the intellect worked?"

In regard to light, I was not long in discovering that my knowledge of its existence was altogether dependent on the particular forms of structure of the eye; for, without these, neither I, nor any other human being, could know anything of light. The knowledge that there was such a thing as sound, depended in like manner on the particular forms of structure of an auditory, or hearing apparatus. So of smell, and taste, both were dependent on special molecular forms of organic structure. And the functions of these special molecular forms of structure were not interchangeable with any other structures of my body. No other member or part of my body but my eye, for example, recognized or made evident the existence of light. And none could hear or recognize the existence of sound but my ears. Nor taste, nor smell, except through particular forms of structure endowed for the performance of these particular functions. But each of these special organic instrumentalities was endowed, in addition, with a sensibility common to all the structures, though of varying intensity. It was, therefore, evident that my body, in virtue of this common sensibility, was a unity, whether in health or disease; and that such a thing as a local disease, or a locally operating remedial agency, was an utter impossibility. In consequence of these facts, to wit, that the existence of matter, force, light, sound, etc., were dependent on particular molecular forms of organic structures, I wrote down the third general law, as follows: "Function is the language or expression of molecular forms of structure behind it."

The next necessary step was to obtain proper conceptions of how or in what manner these molecular forms of structure performed their functions. Were they like the watch, recording the lapse of time on its dial, the interior structures remaining intact or perfect? Or, were they like the candle, performing duty or function at the expense of substance?

The evidence on this point was clear and decisive that their duty was performed at the immediate expense of molecular forms of structure. Thus, the supply of proper food at short intervals, the excreta from the lungs, skin, bladder and bowels, all testify to the molecular changes going on incessantly within; that sleep, or entire repose from duty, at short intervals, of the special senses, was a needful condition of repair. These and many other facts made it evident to my mind that the fourth and fifth general laws were as follows: "Organic or life force, so to speak, stored up in molecular forms of organic structures;" and "For every dynamic result there must be changes of matter."

I felt that thus far all was safe. These laws were absolute; for no exceptions could be found anywhere. And this brought me face to face with the so-called vital force. Was it a fact? or was it a myth? Where was it? and what was it?

Before proceeding to the solution of these questions, it may be well to return to the starting point for a review or summary of settled points. They are: Reign of law supreme;—matter and force persistent;—force correlated;—function the expression of the molecular forms of structure behind it;—force stored up, so to speak, in the molecular forms of structure;—for every dynamic result there are changes of matter.

The next point, then, for solution is "Where is the vital force?" Is it in digestion and assimilation? The conditions for digestion and assimilation to take place are, a living being, proper food to be digested and assimilated to its forms of structure, and introduced into a proper receptacle or stomach. These two processes or functions are performed in the human body by a necessarily complicated apparatus, and at the expense of the molecular forms of structure, the force for which is stored up in the several necessary structures. The ultimate materials, in both food and organic structures, are all inorganic. Certain physical conditions are necessary, as temperature and the inorganic gases of the atmosphere—oxygen and nitrogen. The necessary force is evolved by the decay, by oxidation, of existing molecular forms of structure, as evidenced by the fact that, after loss of molecular forms of structure, the capacity for digestion ceases.* And an indispensable condition of digestion is that the gaseous atmosphere shall be introduced into the living body in an almost unbroken stream at the lungs. There is in the evolution of force a direct or indirect union between an inorganic gas and organic structure. That this is so is evidenced by the fact that the expired gases differ widely in chemical states from the inspired. And again demonstrated, negatively, by cutting off the supply of atmospheric gases, when the chemical changes of matter in such a way as to reproduce the momentarily wasting molecular forms of structure cease. Other chemical affinities come into play, and there is a hasty

* For demonstrative cases, see N. Y. Med. Journal, March, 1871.

retrocession of all organized matter present to simpler chemical states; the correlation of force being heat. Putrefactive decomposition is clearly a physical process, occurring in obedience to known physical laws. But as the reproduction of molecular forms of structure has never been satisfactorily accounted for by any known chemical or physical laws, it has been referred, from the remotest antiquity, to the operation of a peculiar vital or life force. And if it cannot be satisfactorily accounted for, it must still be so considered, for right here is the so-called vital force, if it has any existence at all.

The human stomach, besides being a vessel, as it were, in which a part of the earlier acts of the digestive processes takes place, in the language of physiology, "secretes" an organic principle called "pepsin," which authoritative physiology teaches to be the active principle of digestion. It appears to me that while this statement is true, it does not express the whole truth. My own conclusion in reference to pepsin, as well as all other secretions elaborated by the several viscuses, is that they evolve the force for the reproduction of the molecular forms of structure of the several viscuses themselves; for the main purpose of all organic life is its own preservation and multiplication. And with some of the simpler forms of animal life, as well as most of what are called animals in the vegetable world, the means of reproduction are co-incident with the death of the parent. And thus closely are death and life linked together in the organic world. And in these facts I find a satisfactory explanation of how the momentarily wasting molecular forms of the human body are reproduced. The stomach is demonstrated by physiology to "secrete" an organic principle called "pepsin," whose purpose, it has heretofore been conceded by all investigators, is to carry on the process of digestion. Granting this to be true, it is very evident to me that the statement does not include the whole truth, nor the main purpose of pepsin. Guided by the analogies furnished elsewhere in organic life, its main purpose is the perpetuation of or reproduction of the molecular forms of structure of the stomach itself, completing which, its force is finally merged into and appears as heat.

And it seems to me that, in so far as the processes of digestion and assimilation are regarded as due to the so-called vital force, I have here traced physical force into vital force, synthetically, and vital force into physical, analytically, making the needed circle of the correlation of the physical and vital, and vital and physical forces complete, without finding anything, for the proper understanding of which, it is necessary to refer to the operations of a hypothetical vital force. An organic structure, incapable or not provided with the means of its own perpetuation and multiplication, would soon drop out of existence. And the mistake of histologists has been in not looking to the structures themselves for the means of their own perpetuation, rather than to an imaginary vital force.

In this way my mind was entirely satisfied that whatever modes of force were manifested in my body, to which the term vital was applied, were correlations of the ordinary physical forces, even to the preservation and reproduction of organic forms of structure. As a practical pathologist and therapeutist, the correlation of the physical and so-called vital forces was made further manifest in my ability, by means of therapeutic remedial, and hygienic agencies, to increase or decrease the apparent volume of so-called vital force in the bodies of my patients, and in a measure to control its movements. And further, that upon the accomplishment of the correlation of the physical and so-called vital forces rested the value of remedial agencies in the treatment of so called disease. I say so-called disease, because reasons will be given presently to show that disease as commonly understood is altogether a myth.

But the ultimate mystery of life I did not succeed in clearing up in my investigations.

Personally, I believe in the Bible account of creation. But the act of creation did not of necessity involve the creation of a special or vital force peculiar to life. Nor was it limited as to time. Contrary to the generally received conclusions, I find no foot prints of a Creator in either the matter or force of the human body, limiting my conceptions now to the phenomena of life as manifested by it. But I do find His seal in the marvellous molecular forms of structure evolving the phenomena of life.

Is the faculty of instinct, whether in man or the lower animals, the result of a force which cannot be explained in any other way than by referring it to an unknown and hypothetical vital force?

Instinct, that is, certain actions, by man and animals, performed independently of instruction or experience, without any end in view, but generally tending to the preservation of life and perpetuation of their kind. This I found to be a function depending on molecular forms of structure behind it; measurably perfect at birth, like sight and hearing; and, like all other organic functions, performed at the expense of structure on which it depends. It is precisely analogous to sight and hearing, brought about by physical conditions of matter, and due to modes of physical force; and is, therefore, entitled to the appellation of being vital, only in virtue of its being strikingly manifested by living beings. Instinct differs from the intellectual faculties manifested by man in this, that the molecular forms of structure evolving instinct are measurably perfect at birth, but susceptible of being added to by the incidents and necessities of life—experience; while the molecular forms of structure upon which the mental faculties depend, must be added, so to speak, to the original mass of brain matter at birth, by what is called education; oftener against the will of the recipient than with its consent, even after the stage of adult life has been passed. It is an old proverb that experience is a dear but effectual teacher, whose lessons are not easily forgotten.

And these facts explain intelligibly all of the phenomena of the physical, moral and intellectual life peculiar to different peoples and nationalities on the earth. It makes manifest, for instance, why the Eastern nations so persistently refuse to receive European civilization, arts, science and religion; and why they not unfrequently massacre, *en masse*, their propagandists, as recently witnessed in China. Studied from this point, recent events in Europe become more intelligible; and the causes of the chronic state of anarchy in the Central and South American republics can be understood; as well as the peculiar social, moral and political condition of a part of the people in parts of our own Southern States.

Empirical experience has shown that for the discontinuance of such results, human law is powerless, but a change can be brought about by education, social culture and religion, if patiently and persistently persevered in with the youth only; as no desired results follow such labors with those advanced in life.

It was, therefore, evident to my mind that even the higher intellectual faculties of the human mind presented no evidence of a peculiar vital force not a correlation of the ordinary physical forces, in all that concerns the chemical circulation of matter. That is, again, that neither in matter nor the force which gave it motion, could be traced any foot prints of a Great First Cause.

The sensory, emotional, intellectual and psychological functions, as well as the thermal and mechanical, as incidents, are coincident always with chemical changes in the molecular forms of structure, and never otherwise than in the interest of waste or decay—that is simply motion of matter; and the chemical motion of matter is common alike to organic and inorganic nature; and in it there is certainly nothing peculiarly vital. These movements, like most other effective motion in nature, proceed in secrecy and silence, and therefore escape observation, except by their results.

But HIS Foot Prints—the clear and unmistakable evidence of creative power—vitality worthy of the name—and the ideas associated with it, are seen in the molecular forms of structure evolving these wonderful results. In all organic structures, then, only ordinary inorganic matter is found present; the work of organization performed by the ordinary physical forces; the molecular forms of structure the work of the Great Architect or Creator!

Pushing investigations beyond this point, it will be seen that the whole catalogue of separate and distinct diseases pass out of possible existence; and in their place rises an orderly group of departures from the normal velocities of chemical changes in the molecular forms of structure; and other groups of lost or changed, or changing molecular forms of structure, as evidenced by changed function, together with physical peculiarities of color and contour, and increase or decrease of temperature above or below the physiological standard.

What therapeutic, remedial or hygienic agencies can, or do, do, it is not difficult to determine from these exact ideals of pathology. And it is a striking fact that the greater number only influence motion; and in their operation is again completely demonstrated the correlation of the physical and so-called vital forces. Remedial agencies, promoting or retarding the physical or chemical circulation of matter, afford just, proper and truthful mental conceptions, reflecting back to the mind the facts of therapeutical experiences.

Over forms of structure, it is the evidence of empirical experience through all the past, that pathologists and therapeutists have little, if any, control, except their destruction by surgical or other proceedings. And to losses of molecular forms of structure are due the change in human bodies, which is called old age; for if the molecular forms of structure were maintained intact, without variations from the physiological velocities of changes of matter, or motion, human bodies would never grow old, nor man's faculties become dimmed simply as a result of passing years, or time.

Two cases, coming under my professional notice to-day (March 14, 1871,) may serve to illustrate these general principles and conclusions:

Mrs. D., aged 90. Has lost for ten days past the power of speech. Hearing and vision not so much impaired; suffers no pain; utterly refuses food and medicine, (and she is wise in regard to medicine); sleeps much of the time; passes water twice or thrice in twenty-four hours; has had no passage from bowels for ten days. Apparently recognizes her children and grand children. Has no desire to live. Pulse 30; respirations 7 6-8, per minute. Temperature not ascertained.

V. K., aged 7 months, Large, and fully developed, coughs some; is hoarse; has a flushed face; and is restless; and is druling at the mouth so as to keep his clothing wet on his breast all the time. Passages from bowels fetid; temperature, 100; pulse, 105; respirations, 24. Nurses well, does not throw up. But the parents are anxious; they fear cramp.

No contrast could be more striking than these two cases afford. In the first, function after function has failed, until that of assimilation has been included. It has been demonstrated times without number that behind these changed functions in the aged there exists fibrinous, calcareous, or fatty degenerations of the molecular forms of structure, *i. e.*, loss of molecular forms of structure. Motion in chemical changes has been reduced to its minimum, and the loss of molecular forms proceeding more rapidly; and no doubt physical death is close at hand.

In the second, motion in the interest of waste is slightly in excess of repair, as evidenced by the temperature, breathings, hoarseness and leakage from the mucous surfaces of the mouth, with retention of the results of tissue metamorphosis, or dead matter. In this latter case, a saline purgative, (calcined magnesia, in this instance,) repeated two or three times in as many days, can hardly fail to give exit to the retained results of tissue decay, and thus remove the impediment in the way of repair, with a speedy equilibrium of repair and waste which will be evidenced by returning health, (since realized.)

I therefore conclude, that when the human body is studied, as any other problem in nature is studied, on the known facts concerning its physical organization, the correlation of its hitherto regarded special vital force, or forces, with the ordinary physical forces will be fully demonstrated.

Second, That each histological structure in the act of decay, and the performance of its special function in the general economy of the human body, as a totality, or unity, stores up in one or more of the chemical compounds then formed, the necessary force for its own reproduction and perpetuation, in obedience to chemical and physical law.

Third. That reproduction must, to perpetuate any organic structure, or existence, be in detail, molecule by molecule, and not in bulk, or gross, and at once.

Fourth. That only in the molecular forms of structure of the human body, and not in physical contour, material itself, or chemical motion in material, can the foot prints of a Great First Cause, —a Creator, worthy of adoration, be unmistakably discerned.

Fifth. That its mechanical, chemical, thermal, sensory, emotional, intellectual, and psychological phenomena, are functions simply of its truly marvellous molecular forms of structure, and though differing so widely, these have a material in common,—made of one stuff.

Sixth. That the foregoing conclusions do away with the necessity for referring any of the phenomena of life to a special vital force.

Lastly. That the sensation common to all its structures, demonstrates its unity; and consequently, the impossibility of the existence of any specifically distinct pathological states, and the most urgent necessity for simpler and truer views or ideals of its physiology, dynamics, pathology and therapeutics.

